# Singular Neurosis

**Published:** 1844-02-24

**Authors:** 


### SINGULAR NEUROSIS.

[The following is the detail of a singular affection referable to the reflex nervous system, which was written by the patient himself, an intelligent physician of Indiana, on consulting Professor Dunglison, who has favoured us with the statement, in regard to the history and management of the case.—Ed.]

The singular affection under which I have laboured for about eighteen years, commenced as follows: Having passed a day (I think it was in the month of June) without eating until near night, and being quite hungry, I ate *very freely* of fried ham, with eggs, butter, &c. I soon found that my stomach was oppressed, and feeling stupid, I threw myself on a bed, and in a short time was in a dose, from which I was suddenly awakened by a shock resembling very much that produced by electricity, but of much greater force than is given by an ordinary electric machine. If a horse-pistol had been fired off in my stomach, the jar, or concussion, could not have been greater; and at the moment of the shock it seemed as if flashes of fire passed from my eyes, and the sound of a clap of thunder rang through my ears. I ought here to remark, that previous to this attack, I had long suffered with debility of my stomach, connected with sour eructations, constipated bowels, &c., indicating the approach of dyspepsy, a disease which had been almost hereditary in my mother's family. From the period of the first attack of my disease, to the present time, I have not passed any three nights together without having from 5 to 20 shocks, before I can get to sleep. It is only when in the act, so to speak, of falling asleep, that I have these shocks; and just at the moment when I cease to be conscious, something like a flash of lightning passes, it seems to me, from the diaphragm, through my stomach and up the œsophagus, and discharges itself through my mouth. The commencement, and termination of the shock are inseparable; and resemble in that particular a flash of lightning. Sometimes, instead of the shock passing out at my mouth, it sprangles over my face, following the course of the nerves, and imparts a sensation as if I were stricken with a dozen whip lashes on the face at the same instant; but the pain produced is gone with the shock. Not being conscious at the moment of attack, it is impossible for me to describe accurately my sensations; but I am inclined to believe there is in every instance a spasmodic action of the diaphragm similar to what takes place in singultus, and often a spasmodic action of the muscles of my face is produced by the shock, but this spasm is only for a moment, and not accompanied with any pain whatever. The flashing of fire, or rather pencils of light, from my eyes, and the thunder-crash in my ears, have disappeared; and I now suffer much less from this singular disease than I did formerly. On some rare occasions, the direction of the shock is changed, and passes from my stomach to one or other of my feet, but rarely to my arms. When in bed, the accidental touch of any part of my spine by a bedfellow, produces when I am dozing an immediate shock or spasm. My general health is moderately good, and my appetite unfailing. Distension of my stomach, either from food or drink, always increases the force and frequency of the spasms or shocks. So far as I have made a trial of remedies, I have found no relief from any, except tonics, and blisters on my spine; these for a short time have afforded me relief.

